# Hospital Dispensers and the Insurance Act

**Published:** 1912-05-18

**Authors:** 


### Hospital Dispensers and the Insurance Act.

At a meeting of English pharmacists held at 17 Bloomsbury Square, W.C., on Wednesday, May 8, the English National Pharmaceutical Committee on Insurance was elected. This Committee will consist of the seventeen members of the Standing Pharmaceutical Committee on Insurance, together with ten others who have been elected, one for each of the ten divisions into which the country of England has been divided by the Registrar-General. In addition to the business for which the meeting was specifically convened, the following resolution was passed :

"That this meeting of English pharmacists, assembled for the purpose of setting up the National Pharmaceutical Committee on Insurance (England), expresses general agreement with the demands of the medical profession for adequate remuneration for professional services under the Insurance Act, but protests against the claim that dispensing, as hitherto, should be done, or arranged for, by the medical practitioner, should he so desire, and then paid for at the scale of tariffs agreed upon by pharmacists. That this meeting considers this additional claim to be a demand, not for remuneration for professional medical services, but for additional remuneration from the profit on the supply of medicines, drugs, and appliances for insured persons, and further protests that such a claim is inconsistent with the specified pharmaceutical functions provided for by Clause XV. of the Act."

This resolution was strongly advocated by Mr. Glyn-Jones, M.P.

